# Supplementary material for: Apple latent spherical virus structure with stable capsid frame supports quasi-stable protrusions expediting genome release
Source: Commun Biol. 2020 Sep 4;3:488. doi: 10.1038/s42003-020-01217-4 (PMC7474077; doi:10.1038/s42003-020-01217-4)
Supplement: Supplementary file 1 — Supplementary Information [file 42003_2020_1217_MOESM1_ESM.pdf]

Supplementary Information

Apple latent spherical virus structure with stable capsid frame supports quasi-stable protrusions expediting genome release

Hisashi Naitow[1, #], Tasuku Hamaguchi[1, #], Saori Maki-Yonekura[1], Masamichi Isogai[2], Nobuyuki Yoshikawa[3] and Koji Yonekura[1, 4, *]

[1] Biostructural Mechanism Laboratory, RIKEN SPring-8 Center, 1-1-1 Kouto, Sayo, Hyogo 679-5148, Japan

[2] Faculty of Agriculture, Iwate University, Morioka, Iwate 020-8550, Japan

[3] Agri-Innovation Center, Iwate University, Morioka, Iwate 020-8550, Japan

[4] Advanced Electron Microscope Development Unit, RIKEN-JEOL Collaboration Center, RIKEN Baton Zone Program, 1-1-1 Kouto, Sayo, Hyogo 679-5148, Japan

[#] These authors contributed equally to this work.

[*] To whom correspondence should be addressed.

E-mail: yone@spring8.or.jp

Supplementary Figures

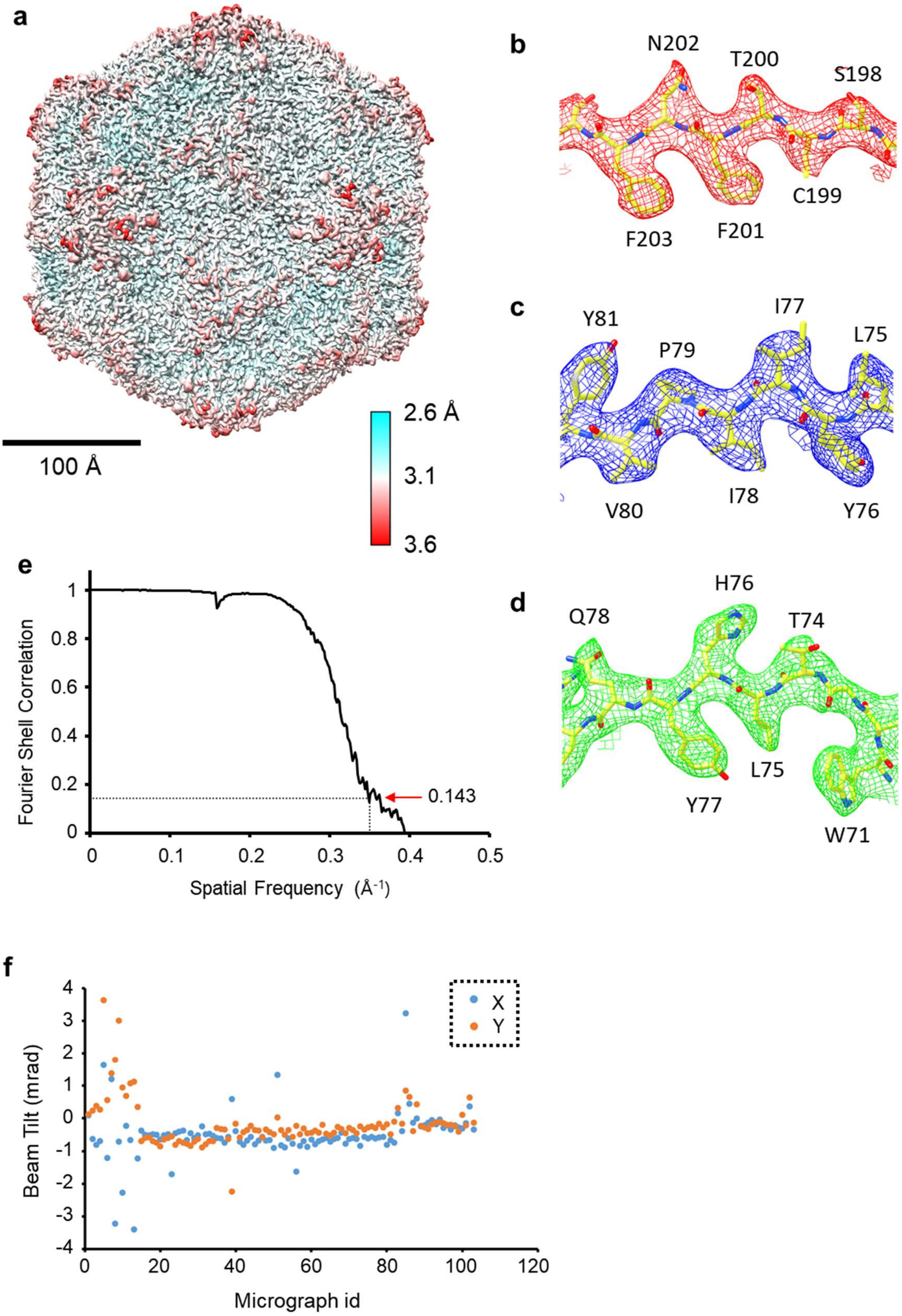

Supplementary Figure 1 Cryo-EM structure of ALSV. (a) Three-dimensional reconstruction. Local resolutions are calculated from unfiltered half-maps and shown in multi-colors according to the scale bar. See also Supplementary Movie 1. (b, c, d) Part of density maps overlaid with atomic models built on the 3D map in (a). Covers residues 198 to 204 in Vp25(b), 75 to 81 in Vp20 (c), and the lower 71 to 78 in Vp24 (d). Contour at 2.5 δ. (e) Fourier shell correlation (FSC) curve. The resolution of the structure is estimated to 2.67 Å based on the gold standard Fourier shell correlation (FSC) criteria [1], where the FSC between two volumes, each independently generated from half the data set, drops to 0.143. (f) Fluctuation of beam tilts in every micrograph.

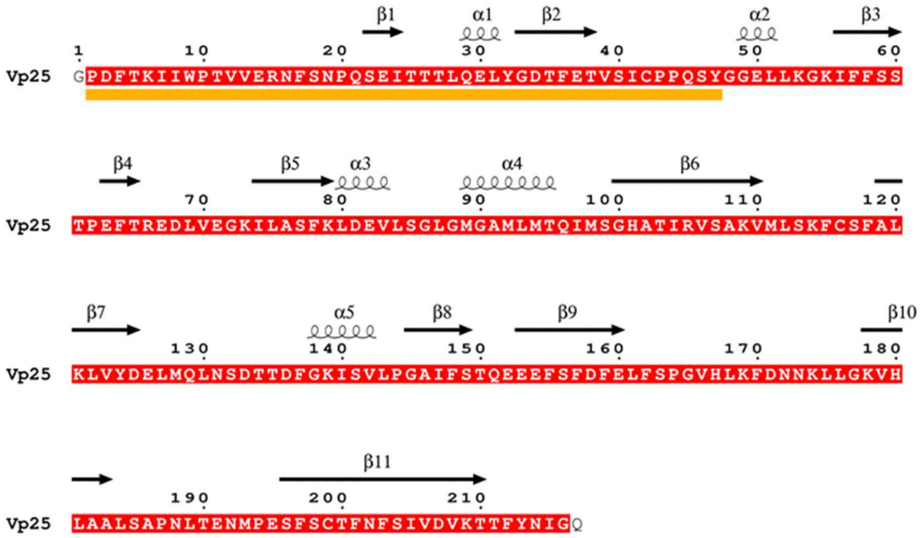

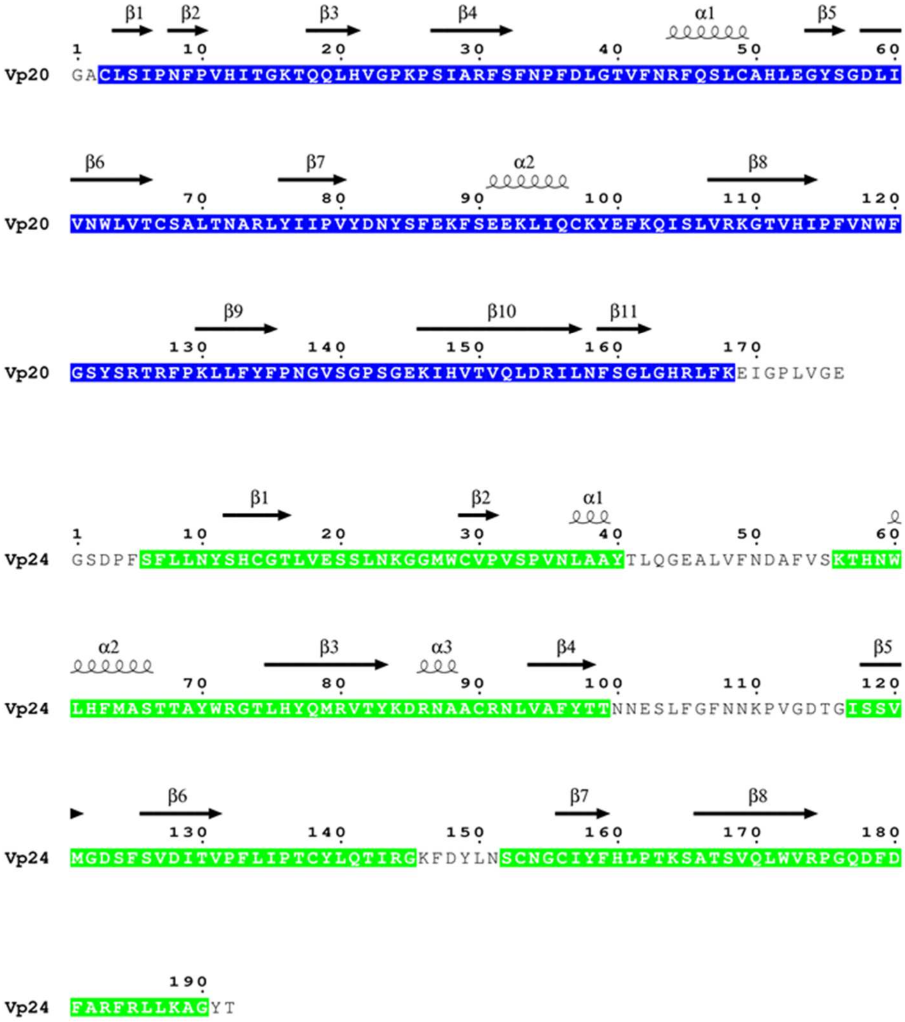

Supplementary Figure 2 Sequence of ALSV with α-helices and β-strands marked. Residues overlaid on red (Vp25), blue (Vp20) and green boxes (Vp24) are modeled on the cryo-EM map, and others are missing in the map. The N-terminal extension of Vp25 is indicated with an orange line as in Fig. 1(c). Drawn by ESPript3 [2].

```
ALSV        GPDFTKIIWPTVVERNFSNPQSEITTTLQELYGDTFETVSICPPQSYGGELLKGKIFFSS 60
CPMV        -------------------------------MEQNLFALSLDDTSSVRGSLLDTK--FAQ 27
TRSV        -------------------------------------AVTVVPDPTCCGTLS-------- 15
                                                 ::::    :  * *

ALSV        TPEFTREDLVEGKILASFKLDEVLSGLGMG---AMLMTQIMSGHATIRVSAKVMLSKFCS 117
CPMV        TRVLLSKAMAGGDVLLDEYLYDVVNGQDFRATVAFLRTHVITGKIKVTATTNISDNSGCC 87
TRSV        --FKVPKDAKKGKHLGTFDIRQAIMDYGGLHSQEWCAKGIVNPTFTVRMH--APRNAFAG 71
                  :    *. *    : :.: . .         . ::.   .:        .  .

ALSV        FALKLVYDELMQLNS-DTTD---FGKISVLPGAIFSTQEEEFSFDFELFSPGVHLKFDNN 173
CPMV        LMLAINSGVRGKYST-DVYTICSQDSMTWNPG-----CKKNFSFTFNPNPCGDSWSAEMI 141
TRSV        LSIACTFDDYKRIDLPALGNECPPSEMFELPTKVFMLKD----------ADVHEWQFNYG 121
            : :    .   : .          ..:   *       .                .  :

ALSV        KLLGKVHLAALSAPNLTENMPESFSCTFNFSIVDVK--TTFYN----------------- 214
CPMV        -SRSRVRMTVICVSGWTLSPTTDVIAKLDWSIVNEKCEPTIYHLADCQNWLPL-NRWMGK 199
TRSV        ELTGHG--------------------LCNWANV--ATQPTLYFFVASTNQVTMAADWQCI 159
               .:                       ::: *      *:*

          Vp25←|→Vp20
ALSV        --IGQGACLSIPNFPVHITGKTQQLHVGPKPSIARFSFNPFDLGTVFNRFQSLCAHLEGY 272
CPMV        LTFPQGVTSEVRRMPLSIGGGAGATQA--------------FLANMPNSWISM---WRYF 242
TRSV        V----------------------TMHVDMGPVIDRFELNPTM--TWPIQLGDTFAIDRYY 195
                                     :.                 .      .     . :

ALSV        SGDLIVNWLVTCSALTN----------ARLYIIPVYDNYSFEK-----------FSEEKL 311
CPMV        RGELHFEVTKMSSPYIK----------ATVTFLIAFGNLSDAFGFYESFPHRIVQFAEVE 292
TRSV        EAKEIKLDGSTSMLSISYNFGGPVKHSKKHAISYSRAVMSRNLGWSGTISGSVKSVS--S 253
             ..        .    .              :       *

ALSV        IQCKYEFKQISLVRKGTVHIPFVNWFGSYSRTRFPKLLFYFPNGVSGPSGEKIHV----T 367
CPMV        EKCTLVFSQQEFVTAWSTQ---VNPRTTLEADGCPYLYAIIHDSTTGTISGDFNL----G 345
TRSV        LFCTASFV----IFPWECEAPP----------TLRQVLWGPHQIMHGDGQFEIAIKTRLH 299
              *.   *     :     .                 :     :   *    .: :
```

```
                                     Vp20←|→Vp24
ALSV            VQLDRILNFSGLGHRLF----KEIGPLVGEGS-DPFSFLLNYSH--CGTLVESSLNKGG-419
CPMV            VKLVGIKDFCGIGSNPGIDGSRLLGPVCAEAS-DVYSPCMIAST--P------------- 389
TRSV            SAATTEEGFGRLGILPL------SGPIAPDAHVGSYEFIVHINTWRPDSQVHPPMFSSSE 353
                       .*  :*           **:  :.  . :.  :  .

ALSV            -------MWCVPVSPVNLAAYTLQGEALVFNDAFVSKTHNWLHFM--------ASTTAYW 464
CPMV            ---------PAPFSDVTAVTFDLING-----KITPVGDDNWNTHIYNPPIMNVLRTAAWK 435
TRSV            LYNWFTLTNLKPDANTGVVNFDIPGYIHDFA------SKDATVTLASNPLSWLVAATGWH 407
                           * : .  . : : .             .:    :          ::.:

ALSV            RGTLHYQMRVTYK----------------DRNAAC-RNLVAFYTTN--NESLFGFNNKPV 505
CPMV            SGTIHVQLNVRGA----------------GVKRADWDGQVFVYLRQSMNPESYDARTFVI 479
TRSV            YGEVDLCISWSRSKQAQAQEGSVSITTNYRDWGAYWQGQARIYDLRRTEAEIPIFLGSYA 467
                 * :.  :                         *   . . .*  .  : .

ALSV            GDTGISSVMGDSFSVDITVPF-----------LIPTCYLQTIRG------------KFDY 542
CPMV            SQPGSAM---LNFSFDIIGPNSGFEFAESPWANQTTWYLECVATNPRQIQQFEVNMRFDP 536
TRSV            GATPSGA-LGKQNYVRISIVN------------------------AKDIVALRVCLR--- 499
                .    .     .  . *                                       :

ALSV            LNSCNGCIYFHLPTKSATSVQLWVRPGQDFDFARFRLLKAGYT--------    585
CPMV            NFRVAGNILMPP-----FPLSTETPPLLKFRFRDIERSKRSVMVQHTATAA    582
TRSV            ----------------PKSIKFWGRSATLF---------------------    513
                                   :.        *
```

Supplementary Figure 3 Sequence alignment of ALSV, CPMV and TRSV by Cluster Omega [3]. Protein subunits of ALSV and CPMV are separated by vertical lines in magenta. Red and blue letters represent α-helices and β-strands, which do not match well unless aided by 3D structure fitting (see text), and ".", ":" and "*" indicate weakly, strongly, and fully conserved residues, respectively. The N-terminal extension of Vp25 are indicated as in Supplementary Fig. 2.

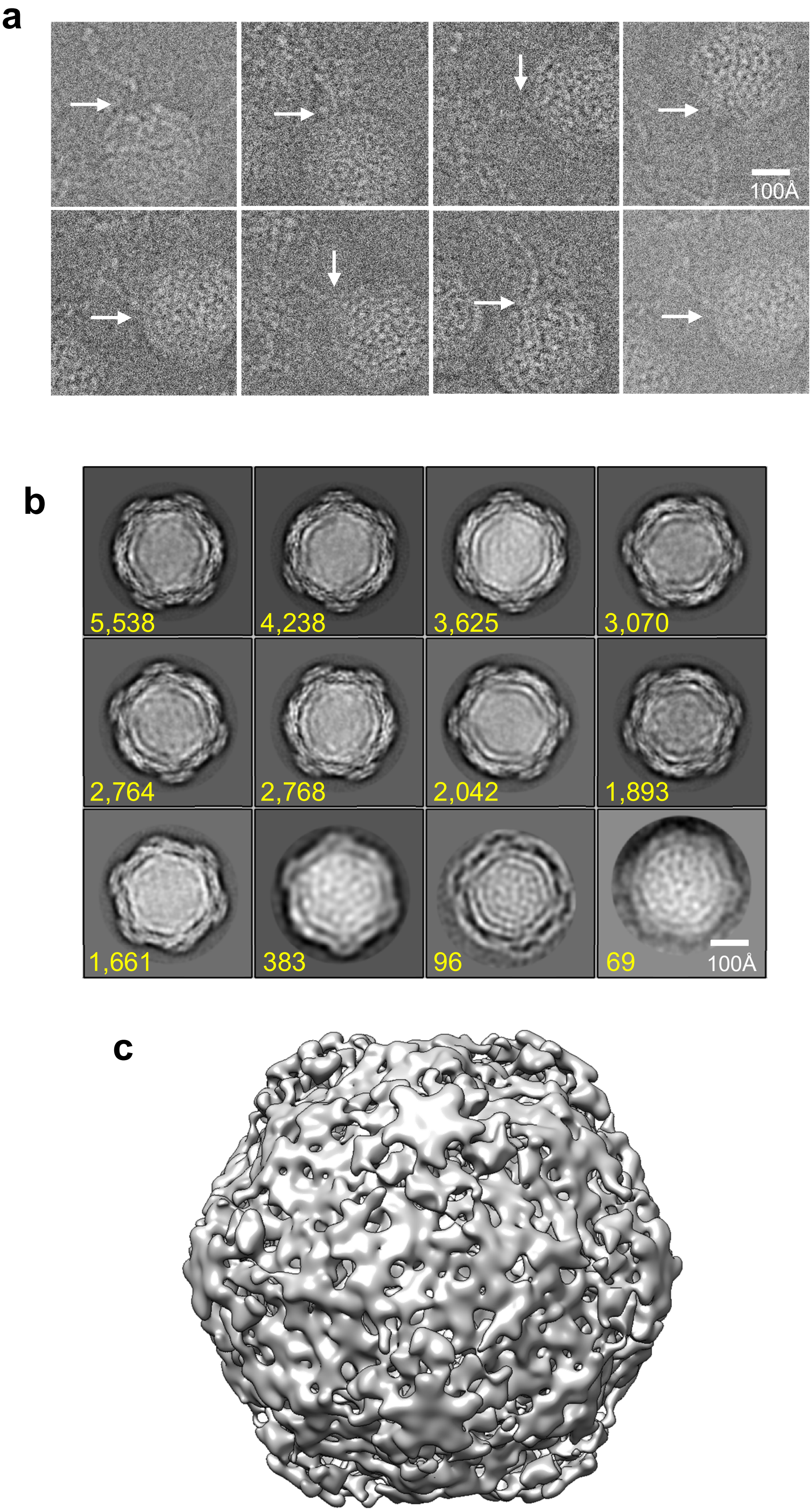
a
100Å
b
5,538
4,238
3,625
3,070
2,764
2,768
2,042
1,893
1,661
383
96
69
100Å
c

Supplementary Figure 4 Two-dimensional images of virus particles (a) Typical images contributing to the 2D class averages in Fig. 4c. Arrows indicate points where RNA chains exit the capsid. (b) Top 12 of 2D class averages from a larger dataset of particle images collected manually and automatically (see Section 4.6 in Materials and Methods). Numbers of particle images contributed to the corresponding class averages are shown at the bottom. The dataset was subjected to 2D classification independently with an option of "Ignore CTFs until first peak". No clear defects are observed in the 2D class averages. (c) Three-dimensional reconstruction without symmetry enforcement. Applied after 3D classification, but not 3D auto refinement.

Supplementary Movie 1 A 3D movie of the ALSV structure colored in local-resolution representation (see also Supplementary Fig. 1a). Density of the internal genome does not appear at this display level.

References

1. Chen, S. *et al.* High-resolution noise substitution to measure overfitting and validate resolution in 3D structure determination by single particle electron cryomicroscopy. *Ultramicroscopy* **135**, 24–35 (2013).
2. Robert, X. & Gouet, P. Deciphering key features in protein structures with the new ENDscript server. *Nucleic Acids Res.* **42**, W320–W324 (2014).
3. Zimmermann, L. *et al.* A Completely Reimplemented MPI Bioinformatics Toolkit with a New HHpred Server at its Core. *J. Mol. Biol.* **430**, 2237–2243 (2018).
