# Supplementary material for: Apple latent spherical virus structure with stable capsid frame supports quasi-stable protrusions expediting genome release
Source: Commun Biol. 2020 Sep 4;3:488. doi: 10.1038/s42003-020-01217-4 (PMC7474077; doi:10.1038/s42003-020-01217-4)
Supplement: Supplementary file 2 — Description of Additional Supplementary Files [file 42003_2020_1217_MOESM2_ESM.pdf]

Descriptions of Additional Supplementary Files

Supplementary Movie 1 A 3D movie of the ALSV structure colored in local-resolution representation (see also Supplementary Fig. 1a). Density of the internal genome does not appear at this display level.

Supplementary Data 1: Source file for the graph in Figure 4e.
